# Evaluation of the Ultima Genomics UG 100 sequencer for low-cost, high-sensitivity metagenomic pathogen detection from cerebrospinal fluid

**DOI:** 10.1128/spectrum.01874-25

**Published:** 2026-03-04

**Authors:** Ryan C. Shean, Keith D. Tardif, Alexandra Rangel, Jacob Dutrschi, David Bogumil, Alix Cruse, Samm Hernandez, Nika Iremadze, Sarah Pollock, Doron Lipson, Benjamin T. Bradley

**Affiliations:** 1 Department of Pathology, University of Utah 161530, Salt Lake City, Utah, USA; 2 ARUP Laboratories 33294, Salt Lake City, Utah, USA; 3 Ultima Genomics 703709 , Fremont, California, USA; Kwame Nkrumah University of Science and Technology, College of Health Sciences, Kumasi, Ghana

**Keywords:** next-generation sequencing, novel diagnostics, mNGS, clinical metagenomics, metagenomics

## Abstract

Clinical metagenomic next-generation sequencing (mNGS) is a diagnostic tool allowing near-universal pathogen detection directly from clinical specimens. Despite promising clinical data, broad adoption of mNGS has been hindered by high cost and reduced sensitivity relative to targeted nucleic acid amplification tests (NAATs). Recently, Ultima Genomics introduced the UG 100 NGS platform which advertises 10 billion reads per $2,400 sequencing wafer. By lowering costs and improving sequencing depth, the historical value proposition of mNGS may be improved. This study evaluates the UG 100 sequencer’s ability to generate reads for metagenomic pathogen detection from cerebrospinal fluid specimens. Ultima reads demonstrated 93% (26/28) positive agreement with orthogonal test results and 63% (10/16) negative agreement against a syndromic panel for meningitis and encephalitis. Near full-length genomes were recovered for three organisms (human herpesvirus-1 [HSV-1], *Streptococcus pneumoniae*, and *Haemophilus influenzae*), with the ability to detect putative antimicrobial resistance genes for *H. influenzae*. Recovery of *Borrelia burgdorferi* reads (6.1 reads per million [RPM] and 9.03 RPM) was achieved from clinical samples with late cycle threshold values (39.7 and 43.0, respectively). Limit of detection (LoD) studies demonstrated detection of HSV-1 and *S. pneumoniae* reads at concentrations of 50 genomes/mL each, which is below the reported LoD for the orthogonal NAATs used in this study. Reducing sequencing costs and improving the analytical sensitivity remove two major hurdles for mNGS adoption by clinical laboratories. While these results are preliminary, they demonstrate a future in which mNGS may be more widely implemented.

**IMPORTANCE:**

Clinical metagenomic next-generation sequencing has struggled to gain wider adoption for nearly a decade, due in part to its high cost and reduced performance versus targeted molecular assays. This study demonstrates the ability of the UG100 sequencing platform to reduce per-base metagenomic sequencing costs while producing reads that maintain high positive agreement with existing molecular assays. Further improvements to cost and analytical performance may shift clinical metagenomics from an expensive test of last resort to a front-line diagnostic for identifying infections.

## INTRODUCTION

Clinical metagenomic next-generation sequencing (mNGS) is a diagnostic methodology that attempts to sequence millions of nucleic acid fragments in parallel from clinical samples to detect pathogens (1). This technique has been shown to have several advantages over traditional microbiologic methods. One such advantage is the ability to provide “hypothesis-free” detection for a range of pathogens (bacteria, viruses, fungi, protozoa, and eukaryotic parasites) without explicit inclusion in the ordering physician’s differential (2).

While initially met with enthusiasm, the broad adoption of mNGS has been hindered by technical and financial limitations. One of the most well-studied applications, cerebrospinal fluid (CSF) mNGS has shown a clinical impact in 28%–57% of suspected meningitis and encephalitis (M/E) cases (3–7). This variability stems partly from differences in how clinical significance is defined, as well as from the reduced sensitivity of current mNGS methods as compared to targeted molecular assays like real-time PCR (3). Financially, mNGS adoption is limited by the high testing cost and the restricted availability of the assay, which is offered mainly through select reference laboratories and companies. To address these issues, many laboratories have implemented stewardship strategies to prioritize testing for individuals with the highest pre-test probability of actionable results (8). If mNGS is to be broadly used as a diagnostic method, testing costs must be reduced and assay sensitivity improved.

Recently, Ultima Genomics (Fremont, CA, USA) introduced the UG 100 sequencer—a novel sequencing platform designed to reduce per-base sequencing costs. Each $2,400 sequencing wafer can produce approximately 10 billion reads (9, 10). This technology is similar to existing platforms (e.g., Illumina), which use sequencing-by-synthesis technology to generate reads. However, cost reductions are achieved through a proprietary reagent delivery platform and utilization of mass-produced sequencing wafers in contrast to machined flow cells. The sequencing process primarily uses unlabeled and non-terminated nucleotides for imaging, improving the signal-to-noise ratio compared to the fluorescently labeled nucleotides employed by other systems (9).

This technology has previously been evaluated for high-throughput applications in human genomics such as single-cell RNA-seq (11), whole transcriptome sequencing (12), and cell-free circulating tumor DNA detection (13). Reducing the sequencing cost of mNGS has the potential to increase the adoption of clinical metagenomics. For example, if the cost, speed, and simplicity of metagenomic testing could approach that of syndromic testing by multiplex PCR panels, the historical use of mNGS as a “test of last resort” may shift to earlier in the diagnostic process. As a result, mNGS may demonstrate better clinical performance and lead to new testing algorithms.

This proof-of-concept study was designed to evaluate the UG 100 sequencing platform for generating reads suitable for metagenomic pathogen detection from clinical CSF samples. Performance characteristics examined included positive and negative agreement versus orthogonal results, relative pathogen nucleic acid recovery, and limit of detection (LoD).

## MATERIALS AND METHODS

### CSF sample preparation

Residual CSF specimens from routine clinical testing performed at a national reference laboratory were selected to cover a range of possible pathogens responsible for M/E. All positive samples required at least one positive test result from a laboratory-developed test or FDA-cleared assay, and samples were selected to cover a variety of orthogonal methods, including samples that were detected near the limit of detection of the orthogonal method. CSF samples negative for all targets on the FilmArray Meningitis/Encephalitis Panel (BioFire, Salt Lake City, UT, USA) were selected to serve as negative controls and matrices for spike-in studies.

Five hundred microliters of CSF specimen was bead beat on a FastPrep24 using Lysing Matrix E (MPBio, Santa Ana, CA, USA) at 6.0 M/S for 40 s. After centrifugation, DNA was extracted from 200 µL of the supernatant using the Perkin Elmer Chemagen MSM1 nucleic acid extraction platform and Chemagic Viral NA/gDNA Kit (PerkinElmer, Springfield, IL, USA), eluting in 50 µL of elution buffer.

Nucleic acid enrichment was performed using the NEBNext Microbiome DNA Enrichment Kit (New England Biolabs, Ipswich, MA, USA) according to the manufacturer’s instructions. DNA extract (40 µL) was combined with 160 µL of MBD2-Fc-bound magnetic beads and 1× binding buffer. Unbound microbial DNA was put through bead cleanup using Agencourt AMPure magnetic beads (Beckman Coulter, Indianapolis, IL, USA). Microbial DNA was eluted in 50 µL of TE.

### LoD testing

Patient CSF specimens positive for human herpesvirus-1 (HSV-1) or *Streptococcus pneumoniae* were used for spiking and were quantified in a published real-time PCR assay for *S. pneumoniae* or an unpublished assay for HSV-1 (ELiTech, Silicon Valley, CA, USA) from a standard curve generated using known concentrations of plasmid DNA (14, 15*)*. HSV-1 assay primers (HSV1-L1 [AATAAATCAGGGAGTTGTTCGGTCATAAGC] and HSV1-E2 [AATAAATCATCGGAACGCACCA*CACAAAAG]) and probe (HSV1-DSQ-AP593 [G*TAGTTGGTCGTTCGC]) were used at 0.3 µM and 0.2 µM, respectively. Super A or super G (*) bases were used to optimize primer and probe design. DNA was amplified with the QuantStudio 12k Flex Real-Time PCR System (ThermoFisher) using the following conditions: 95°C for 10 min followed by 45 cycles of 95°C for 10 s and 55°C for 45 s. Fluorescence was read after each of the 45 cycles. Pooled CSF patient samples that were negative in the BioFire Meningitis/Encephalitis Panel were spiked with pathogen nucleic acid at 500,000 genomes/mL. Tenfold serial dilutions to 5 genomes/mL were created for assessing sensitivity, and each dilution was extracted once for library preparation and sequencing.

### Ultima Genomics sample prep and sequencing

DNA samples were prepared as libraries following Ultima Genomics PCR-Free WGS Library Preparation Protocol (P00014) with a varying input of 10–20 ng of DNA from each sample. Due to the low genomic material of the samples, preparation deviated from the protocol by diluting the adapter input 1:10 prior to adapter ligation. The next libraries were PCR-amplified using Ultima Genomics Library Amplification Kit v4.0 Native User Guide (P00068) with 15 cycles of PCR instead of the recommended 7 cycles.

After library prep, samples were batched to collect approximately 250M reads per sample, corresponding to 20 specimens including a positive human genome control HG002 (Coriell nucleic acid ID: NA24385) per wafer. Next, the samples were sequenced on the UG 100 Sequencer using UG Baseline 1.5 Sequencing Chemistry. This chemistry generated single-end 300 bp reads using the sequencing recipe UG_116cycle_Baseline_1.5.3.2.

### Bioinformatics analyses

All non-amplifying UG primers, adapters, and barcodes were trimmed on the UG 100 server using the Ultima Genomics in-house trimmer software. Reads were then aligned to the human genome assembly GRCh38/hg38 using BWA MEM (16). Reads not aligned to the human genome were extracted from the BAM alignment and converted to FASTQ using samtools v1.11 (17), thereby removing all reads supposedly originating from human DNA. For the purposes of this project, “coverage” refers to the percentage of reference genome bases that have at least one aligned read, and “depth” refers to the average per-base number of reads aligning to the reference.

To assess the suitability of UG 100 sequencing reads for metagenomics, we examined positive and negative agreement and limit of detection. For positive agreement, non-human reads from each sample were manually aligned to a reference genome of the orthogonally confirmed pathogen using Geneious Prime 2025.0.3 (https://www.geneious.com/), and reads were manually verified with BLAST searches against RefSeq and NT (18). Additional k-mer-based metagenomic analysis of samples was also performed with Kraken2 against the pre-built NT database with default settings (19). Negative agreement was assessed for 16 Biofire M/E panel specimens by comparing targets classified as “Not Detected” on the panel to mNGS results. For this proof-of-concept study, results were categorized as discordant if a Biofire “Not Detected” target was observed at ≥3 reads per million (RPM) or above the mean background RPM signal of the negative controls, whichever was greater. Antimicrobial resistance genes were examined using CARD and manually confirmed with BLAST (20).

## RESULTS

### Positive agreement, negative agreement, and sequencing metrics

UG 100 sequencing produced reads aligning to the known pathogen in 93% (26/28) of positive specimens, which included 17 unique organisms (Table 1). Of the two samples which failed detection, one (CSFU1-08) was due to a sequencing failure (no usable reads generated). In the other sample (CSFU2-13), no reads corresponding to the known pathogen (HHV-6) were identified. For positive samples, on a per-sample basis, normalized pathogen RPM ranged from 2.83 to 15,746. Overall genomic coverage of pathogen genomes ranged from 0.1% to 98.1%, and average sequencing depth (average number of reads per reference base) of pathogens ranged from 0 to 304 reads. Negative percent agreement was 63% (10/16). For six specimens in which all Biofire targets were categorized as “not detected,” manual alignment of mNGS reads to reference sequences identified the presence of at least one panel target (Table S1). This analysis was also repeated with Kraken2, which uses a k-mer-based approach to classify mNGS reads with similar results (Table S2).

**TABLE 1:** Positive agreement between mNGS using Ultima Genomics chemistry and orthogonal results

ID	Known organism	Positive orthogonal test	Ultima result	RPM	Coverage (%)	Depth (mean)	Reference genome	Non-human reads	Aligned reads
CSFU1-02	*Haemophilus influenzae*	Biofire panel positive	Detected	359.80	97.60	9.3	CP007470.1	121,976	62,726
CSFU1-04	Human herpesvirus 6	Biofire panel positive	Detected	3.57	20.40	1.4	NC_001664	86,063	755
CSFU1-08	Cytomegalovirus (HHV5)	LDT NAAT^*a*^ (CT 29.4)	Not detected	0	0	0	NC_006273.2	–^*b*^	–
CSFU1-10	*Coccidioides immitis*	Complement fixation 1:32	Detected	9.80	1.10	0	NW_004504312.1	96,906	1,721
CSFU1-12	*Taenia solium*	IgG ELISA (24 IU [>11 IU is positive])	Detected	145.54	1.00	0	GCA_002082475.1	180,312	23,329
CSFU1-13	*Borrelia* *burgdorferi*	LDT NAAT (CT 39.7)	Detected	6.10	5.40	0.1	NC_012512.1	147,751	1,188
CSFU1-14	*Streptococcus pneumoniae*	Biofire panel positive	Detected	15.56	20.20	0.3	CP053210	78,369	3,038
CSFU1-16	Herpes simplex virus type 1	Biofire panel positive	Detected	23.76	98.10	6.7	NC_001806.2	94,348	4,684
CSFU1-17	*Toxoplasma* *gondii*	LDT NAAT (CT 31.9)	Detected	286.37	7.20	0.2	NC_031467.1	443,146	54,429
CSFU2-04	*Streptococcus agalactiae*	Biofire panel positive	Detected	18	5.40	0.3	GCF_001552035.1	352,793	3,611
CSFU2-06	*Toxoplasma* *gondii*	LDT NAAT (CT 23.7)	Detected	1,819.34	30.00	1.1	GCF_000006565.2	331,030	273,753
CSFU2-07	*Toxoplasma* *gondii*	LDT NAAT (CT 29.1)	Detected	818.17	10.50	0.6	GCF_000006565.2	297,694	162,377
CSFU2-08	*Taenia solium*	IgG ELISA (35 IU [>11 IU is positive])	Detected	6,898.19	5.20	4.7	GCA_001870725.1	2,220,401	1,472,764
CSFU2-10	*Cryptococcus neoformans*	Biofire panel positive	Detected	1,385.00	2.20	3.9	GCF_000091045.1	3,709,025	244,619
CSFU2-13	Human herpesvirus 6	Biofire panel positive	Not detected	0.00	0.00	0	NC_001664	6,299,977	0
CSFU2-14	Herpes simplex virus type 1	Biofire panel positive	Detected	44.89	63.50	13.9	NC_001806.2	383,631	8,610
CSFU2-15	Herpes simplex virus type 2	Biofire panel positive	Detected	35.21	78.40	8.6	NC_001798	299,159	6,196
CSFU2-16	Varicella zoster virus (HHV3)	Biofire panel positive	Detected	7.16	38.90	2.1	NC_001348	124,193	1,271
CSFU2-17	*Candida* species	LDT NAAT (CT 32.0)	Detected	76.12	0.60	0.1	GCF_000182965.3	944,670	14,381
CSFU2-18	*Coccidioides* species	Complement fixation 1:64, immunodiffusion positive	Detected	139.84	0.60	0	GCF_000149335.2	645,354	27,347
CSFU2-19	*Coccidioides* species	Complement fixation 1:32, immunodiffusion negative	Detected	14.33	0.10	0	GCF_000149335.2	5,286,913	4,952
CSFU2-31	*Neisseria* *meningitidis*	Biofire panel positive	Detected	15,746.29	92.80	304	NZ_CP064367.1	2,778,867	2,290,934
CSFU2-32	*Escherichia coli*	Biofire panel positive	Detected	132.88	49.10	1.2	NC_000913.3	89,277	23,726
CSFU2-33	*Haemophilus influenzae*	Biofire panel positive	Detected	20.04	17.50	0.3	NZ_CP085952.1	67,412	3,377
CSFU2-34	Cytomegalovirus (HHV5)	Biofire panel positive	Detected	2.83	2.20	0.1	NC_006273.2	47,154	445
CSFU2-35	*Listeria monocytogenes*	Biofire panel positive	Detected	28.99	1.50	0.2	NC_003210.1	264,753	5,904
CSFU2-36	*Cryptococcus neoformans*	Biofire panel positive	Detected	188.37	1.40	0.3	GCF_000091045.1	833,540	38,297
CSFU2-37	*Borrelia* *burgdorferi*	LDT NAAT (CT 43.0)	Detected	9.03	1.60	0.1	NC_012512.1	229,929	2,076

^*a*^ NAAT, nucleic acid amplification test.

^*b*^ –, no useable reads.

The average number of total reads per sample over two sequencing runs was 190 million (range: 145–345M), while the average number of non-human reads was 979,802 (47,154–6,299,977) with an average quality score of 32.2 (31.7–32.7) (Table S3). The average read length was 258 nucleotides (mean per-sample read length: 221–281). Off-target reads in negative controls matching M/E panel targets are provided in Table S4.

### Limit of detection study

**Fig 1:**
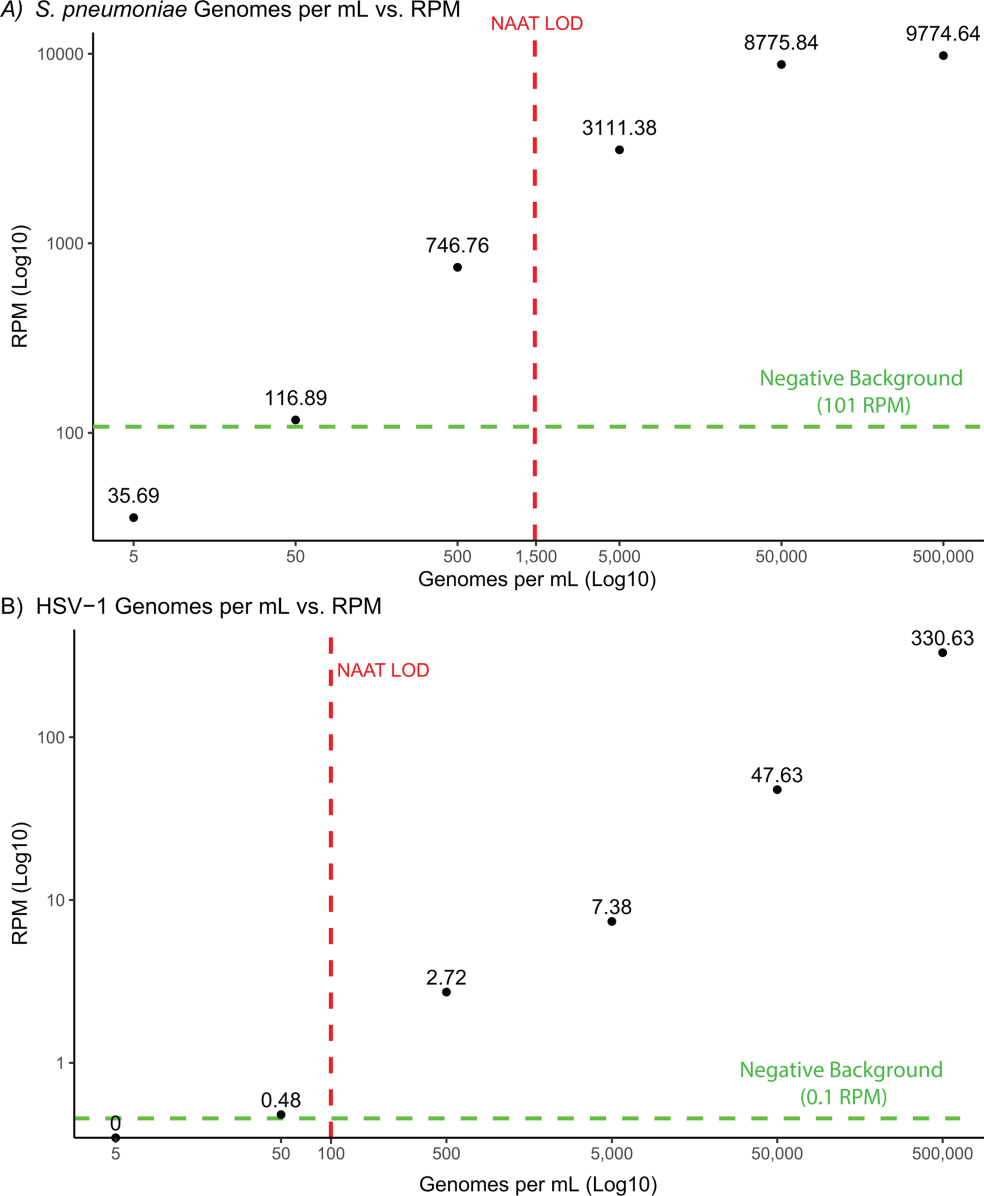
Limit of detection for HSV-1 and *S. pneumoniae* by mNGS on the Ultima UG 100. (**A**) A total of 6,777 reads (35.69 RPM) aligning to the *S. pneumoniae* genome were recovered at 5 genomes/mL below the published LoD of 1,500 genomes/mL for the NAAT (red dotted line). When accounting for background *S. pneumoniae* signal found in negative controls (green dotted line), the LoD increased to 50 genomes/mL but remained below the LoD of the NAAT. (**B**) A total of 77 reads (0.48 RPM) mapping to the HSV-1 genome were recovered at 50 genomes/mL. This is below the 100 genomes/mL LoD for the commercially available NAAT (red dotted line) and above the background signal of the negative controls (green dotted line).

Using a serially log-diluted HSV-1 sample (500,000–5 genomes/mL), 77 non-identical reads (0.48 RPM) mapping to the HSV-1 genome were recovered at 50 genomes/mL, below the 100 genomes/mL LoD for the commercially available nucleic acid amplification test (NAAT) and above the negative background signal. For the serially diluted *S. pneumoniae* samples (500,000–5 genomes/mL), 6,777 reads (35.69 RPM) mapping to the reference genome were recovered at the lowest concentration tested (5 genomes/mL). When accounting for *S. pneumoniae* background signal present in the negative controls, the LoD increased to 50 genomes/mL, still below the published LoD of 1,500 copies/mL for the NAAT (14) (Fig. 1).

### Novel observations

**Fig 2:**
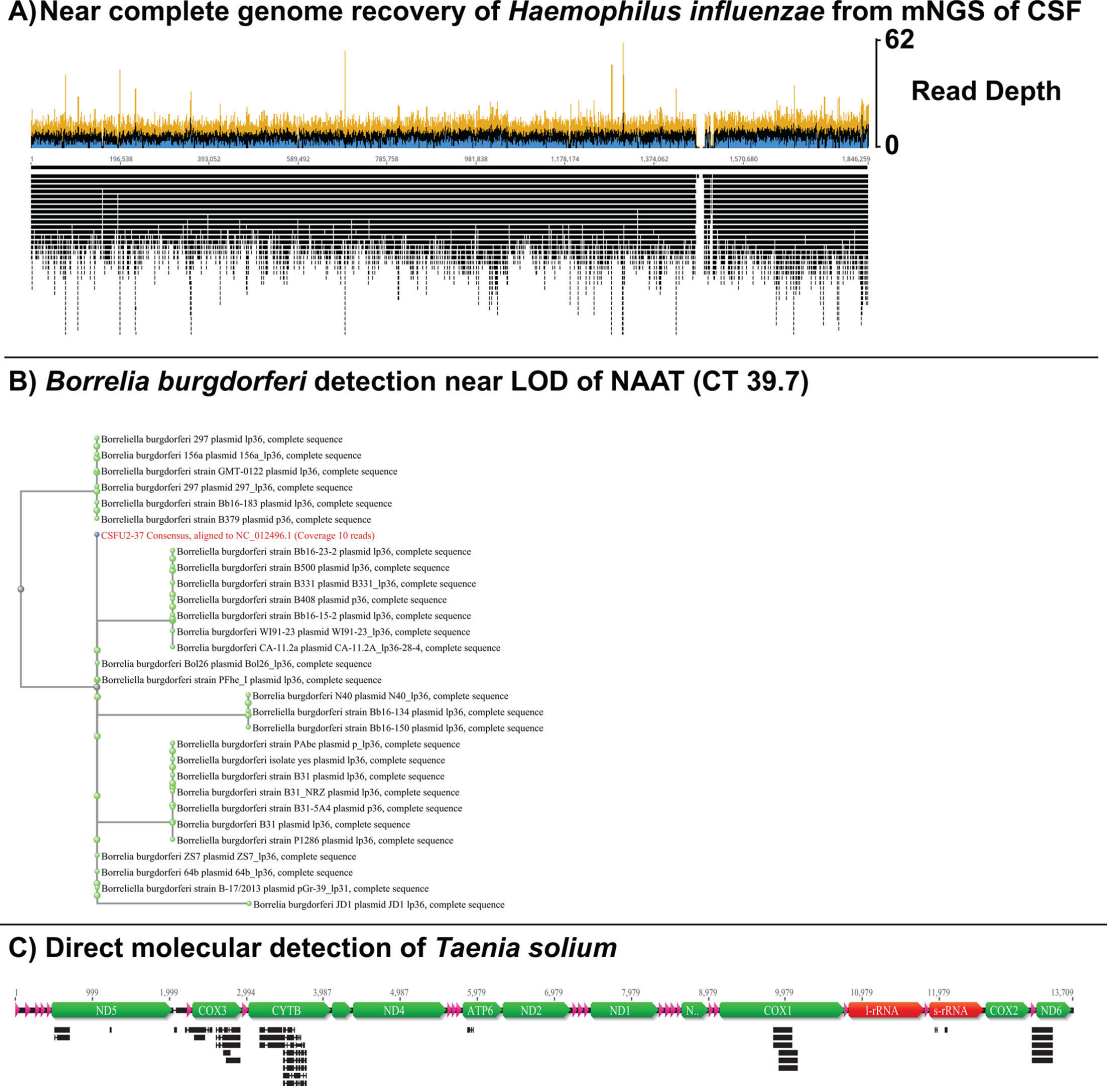
Novel observations following mNGS with Ultima sequencing chemistry. (**A**) Near-complete genome recovery of *H. influenzae* from mNGS of CSF—recovery of a near-complete genome of *H. influenzae* (97.6% genome coverage, average depth 9.3 reads) directly from CSF. (**B**) *Borrelia burgdorferi* detection near LoD of NAAT (CT 39.4)—phylogenetic tree of *B. burgdorferi* using reads recovered (2,076 reads, 6.1 RPM) from a clinical sample near the limit of detection (CT 39.7). (**C**) Direct molecular detection of *Taenia solium—*reads (*n* = 13,409) mapping to specific mitochondrial sequences of *T. solium* recovered from patient with positive serology results.

Near-complete genomes were recovered from three positive samples: CSFU1-02 (*Haemophilus influenzae*; 360 RPM, 97.6% genome coverage, average depth 9.3 reads) (Fig. 2A), CSFU1-16 (HSV-1; 24 RPM, 98.1% genome coverage, average depth 6.7 reads), and CSFU2-31 (*Neisseria meningitidis*; 15,746 RPM, 92.80% genome coverage, average depth 304 reads). Further analysis of the *H. influenzae* sequence identified putative antimicrobial resistance genes, *hmrM* and *lpsA*.

Two samples containing low levels of *Borrelia burgdorferi* DNA (CT value of 39.7 for CSFU1-13 and 43.0 for CSFU2-37) as determined by a laboratory-developed test were positively identified by mNGS at an RPM value of 6.10 and 9.03, respectively (Fig. 2B). Ultima sequencing of two samples (CSFU1-12 and CSFU2-08) with positive serologic evidence for *Taenia solium* infection (IgG ELISA of 24 IU and 35 IU) detected reads corresponding to the *T. solium* genome in both cases, at 146 RPM and 6,898 RPM, respectively. As the publicly available genomes for *T. solium* are poorly annotated, reads corresponding specifically to the *T. solium* mitochondrial sequences were manually confirmed using BLAST (Fig. 2C).

## DISCUSSION

This proof-of-concept study was designed to assess the suitability of the UG 100 sequencing platform for generating reads suitable for metagenomic pathogen detection in cases of M/E. Overall, we found suitable performance characteristics as compared to existing assays with a positive agreement of 93%. In one case (CSFU1-08, CMV), DNA extraction and amplification failed, preventing the generation of sequencing reads and analysis. The other case in which Ultima sequencing failed to detect a pathogen (CSFU2-13, HHV-6), zero reads were recovered that mapped to the HHV-6 reference genome. Basic metagenomic analysis of this sample with Kraken2 revealed many reads (8,454) aligning to HSV-1 in this sample. This sample’s reads were manually aligned to the HSV-1 reference genome (NC_001806), and 7,586 reads were identified. However, these reads were restricted to only a few sites on the genome, suggesting an analytic error (i.e., jackpotting) and showed 100% nucleotide agreement to the HSV-1 isolate used for LoD studies on the same run. Additionally, this sample contained 41,958 reads mapping to *Cryptococcus neoformans*. CSFU2-13 was on the same run as CSFU2-10, which contained a relatively high amount of *C. neoformans* DNA (244,619 reads). Unfortunately, the initial DNA extraction for mNGS depleted the sample, and further analysis with re-sequencing was not possible. As this sample had a positive NAAT for HHV-6 and we recovered zero reads aligning to HHV-6, we suspect a pre-analytic error such as a sample swap with a negative, combined with contamination events exacerbated by physical plate proximity to samples with high pathogen DNA content.

Negative agreement was performed in a subset of 16 samples for which BioFire M/E panel results were available. Analytes present on the panel but reported as “not detected” were used for comparison to mNGS results. The most common discordant detection was for HSV-2 (3/16). After HSV-2, the next most common, with two detections each, were *N. meningitidis*, HSV-1, and *H. influenzae*. All these organisms were present in other samples on the runs at high concentrations. Additionally, as negative agreement was determined by aligning the sample’s non-human reads to the reference genome of each M/E panel target, the bacterial reads likely represent shared bacterial sequence from environmental, skin, container, and reagent components (21). Mitigating the effects of these DNA sources remains a challenge in metagenomic analysis. To illustrate this point, *Cutibacterium acnes* nucleic acid was detected at ≥3 RPM in 94% (15/16) of samples (Table S1). These findings highlight the need for both in-run and between-run background normalization, as metagenomic methods are extremely sensitive to background signal and contamination events.

This study also provided several new observations in how a lower-cost, high-sequencing depth mNGS assay may improve upon existing options. First, we recovered near-complete pathogen genomes from three clinical samples. Significantly increasing the number of reads per sample when performing mNGS opens new avenues of analysis including identification of antimicrobial resistance genes and strain typing for outbreak tracing. We also showed molecular detection of an organism (*T. solium*) for which no FDA-cleared molecular assays are available. Pathogens that have historically required esoteric molecular testing through public health labs or serologic assays for diagnosis may be identified earlier and more precisely with mNGS. Lastly, we demonstrate that our mNGS workflow provides a limit of detection that may surpass that of single target NAATs. This highly sensitive detection of pathogen DNA fragments was evidenced in both contrived samples and two clinical samples positive for *B. burgdorferi* with late cycle threshold values.

### Flipping the diagnostic algorithm and cost comparisons

Historically, mNGS is most likely to be ordered when the traditional diagnostic armamentarium has been exhausted by negative results. Interestingly, many traditional microbiology tests ordered early in the diagnostic workflow also demonstrate low clinical sensitivity but are still used because they are inexpensive and readily available. For example, a single blood culture has between 70%–80% sensitivity and costs between $1–10 (22). While cost is only one of several barriers to broader adoption of mNGS, reducing sequencing expenses could make earlier use of this technology more feasible in certain settings, potentially “flipping” the traditional diagnostic workflow.

In this study, the reagent cost of mNGS on the UG100 compared favorably to the current industry standard. The approximate cost per 50M reads would be $12 using Ultima chemistry, which is 25% of the cost of Illumina NovaSeqX sequencing ($48 per 50M reads) with similar expenses for DNA extraction and library prep. Sequencing run times are also similar between the two platforms (maximum run time ~20 h for ~10B paired-end reads). Combined with sample handling, extraction time, and library prep, this makes it unlikely the UG100 will dramatically improve upon the 48 h turn-around time offered by some reference laboratories. In this study, we targeted $60 in total sequencing costs (or 250M reads per sample, 20 samples per batch, and 10–12B reads per run) and another $40 in reagent costs to bring the total cost to perform mNGS to $100 per sample. While the cost reductions are encouraging, other barriers such as workflow integration, regulatory compliance, bioinformatic overhead, and clinical validation remain significant.

It is important to note that following basic QC and trimming, FASTQ files from the UG 100 sequencing platform can be used with a range of existing bioinformatics tools (FastQC, Geneious, bowtie, MAAFT, Blast, and Kraken2). The ability to “plug and play” with reads from a novel sequencing technology is highly useful as it significantly reduces the bioinformatics and analytic overhead of adopting this novel technology.

### Study limitations and conclusion

Limitations of this study include manual selection of clinical samples, restriction to DNA-based organisms, lack of prospective design, and a lack of robust metagenomic analysis. As in any metagenomic study, reads were recovered to a wide variety of off-target organisms. However, the development and validation of a novel metagenomic analysis pipeline were determined to be beyond the scope of this study. Therefore, manual bioinformatics and basic use of pre-existing tools were used to determine if reads from this sequencer would be usable by other, more sophisticated metagenomic pipelines.

Unfortunately, lowering the cost of metagenomic sequencing is not the sole barrier preventing wider adoption of mNGS. Other considerations such as turn-around time, library preparation time, technical infrastructure, and adjudicating contamination versus true infection remain. For example, inhibition due to high protein and inflammatory cell count and low biomass samples can drastically impact sensitivity. Additionally, with any high-throughput sequencer, data storage and other bioinformatics considerations are non-trivial and add additional cost. Furthermore, there must be sufficient testing volume (estimated in this study at 20 samples per run) to provide an acceptable cost profile.

In summary, the UG 100 sequencing platform offers a new NGS chemistry allowing generation of reads suitable for lower-cost metagenomic pathogen identification. Overall positive agreement was 93% with a LoD that surpassed single-target NAATs. While further bioinformatic refinements are necessary before entering the clinical diagnostic space, this proof-of-concept study illustrates the technical reality needed for first-line mNGS testing of patients may be approaching.

## Data Availability

Sequencing reads generated and analyzed in this study are publicly available in the NCBI Sequence Read Archive under BioProject accession number PRJNA1374438.

## SUPPLEMENTAL MATERIAL

Supplemental tables spectrum.01874-25-s0001.xlsx Tables S1 to S4.

ASM does not own the copyrights to Supplemental Material that may be linked to, or accessed through, an article. The authors have granted ASM a non-exclusive, world-wide license to publish the Supplemental Material files. Please contact the corresponding author directly for reuse.
